# Does club convergence matter? Empirical evidence on inequality in the human development index among Indian states

**DOI:** 10.1057/s41599-023-01518-z

**Published:** 2023-01-19

**Authors:** Ajit Nag, Jalandhar Pradhan

**Affiliations:** Department of Humanities and Social Sciences, National Institute of Technology (NIT), Rourkela, Odisha India

**Keywords:** Economics, Development studies

## Abstract

The Human Development Index (HDI) is recognised as the most commonly used composite index to assess the socio-economic progress of a country. To preserve its pioneering role in development, there has to be a reduction in inequalities and cross-state convergence by adding a sustainable dimension. This paper investigates the convergence hypothesis for the HDI in 36 Indian states and union territories (UTs) from 1990 to 2019. For that purpose, the study used the club convergence technique of Phillips and Sul (2007) and Kernel Density estimates to assess whether states converge towards a single steady-state equilibrium or multiple groups. The paper also considers the relative performance of Indian states and UTs and the comprehension of inter-regional inequality in the HDI by employing the Gini and Theil indices. Using the Phillips and Sul technique, the results reveal that all the states converged into two final clubs (i.e., Club 1 and Club 2). The rate of convergence of HDI is approximately 0.112% for club 1 and 1.135% for club 2. The findings indicate that states with the lowest HDI converge faster than those with higher HDI. The kernel density estimates demonstrate that HDI stratifies, polarises, and becomes unimodal over time, albeit with a common steady state. Further, the Gini and Theil indices suggest a significant decline trend in HDI inequality across the Indian states and UTs from 1990 to 2019. From a policy perspective, the study recommends promoting regional development and reducing inequality, considering the unique convergence paths of the clustering states. The study’s findings could provide the government with a new perspective on attaining “horizontal equity” in HDI across Indian states and UTs.

## Introduction

The United Nations Development Programme (UNDP) introduced the Human Development Index (HDI) in 1990, and it is widely recognised to play a significant role in development when contrasted with national income or economic growth (Klugman et al., 2011; Dervis and Klugman, 2011; Morse, 2014; Javaid et al., 2018). This index not only provides economic performance but also focuses on three essential capabilities: living a long and healthy life, acquiring knowledge, and having access to resources for a decent standard of living, which is the process of expanding people’s choices and plays a crucial role in the development (UNDP, 1990). However, while the fundamental human dimension has stayed constant over time, the indicators used to estimate HDI has changed (Morse, 2014). The high rating demonstrates a country’s ability to improve its health sector and safeguard its inhabitants from various health and survival difficulties (Khazaei et al., 2016; Černák, 2017). It is also a measure of a country’s ability to enhance the quality of its human resources and the quality of life of its citizens in general (Sunarya, 2017; Hakim et al., 2021).

Although worldwide poverty has decreased, increasing inequality is becoming a severe concern in the twenty-first century (WID, 2018; UNDP, 2019). Inequality in health, education, and income across regions and groups deeply affect progressive human development achievement. Many scholars have taken an interest in regional variations and disparity in human development globally (McGillivray and Pillarisetti, 2004; Decancq et al., 2009; McGillivray and Markova, 2009; Chanda and Kabiraj, 2020). Inequalities in human development are primarily a result of disparities in performance across areas and groups in terms of access to essential services such as education, health, and infrastructure (Niranjan, 2020). Inconsistency in HDI convergence within and across countries suggests disparity within and between them, eroding political stability, social interests, and violence (Goswami et al., 2021). As a result, equity is crucial for human development to achieve various sustainable development goals in the long run (Comim et al., 2008). Therefore, reducing inequality and evaluating the impact of targeted development programmes should be the primary concerns in every human development strategy (Reddy et al., 2022).

As the present COVID-19 epidemic demonstrates, health resources and services discrepancies profoundly impact human life and death, jeopardising the sustainability of human development. India has witnessed a dramatic increment in COVID-19 cases and deaths (Sarkar et al., 2020; Kar et al., 2021). Additionally, it has wreaked havoc on the education sector, which is an essential gauge of a country’s economic destiny (Jena, 2020; Kapasia et al., 2020). As a result, it is vital to account for differences in human development when assessing a country’s or region’s level of human development.

Most of the literature on the factors affecting India’s level of human development is based on the classical econometrics approach. Dholakia (2003) studied patterns of regional disparity in India’s economic and human development and the direction of their causality for the period 1981–2001; and found a causal relationship between them. Additionally, he revealed that while inequality in per capita net state domestic product (NSDP) did not significantly decrease, the HDI showed a fall in inequality in the major states. Similarly, for the same period, Ghosh (2006) studied the convergence of the 15 major states of human development in India; and he observed that despite significant differences in per capita income across the states, there is evidence for regional convergence in human development. In addition, some studies in India have evaluated convergence analysis of the HDI using beta and sigma convergence, and their outcomes are similar to the previously mentioned studies (Mukherjee and Chakraborty, 2007, 2011; Roy, 2012; Banerjee and Kuri, 2015).

Despite several analyses of regional economic development and differences in central and state spending, the answer to economic sustainability at the aggregate level remains to be determined. Das et al. (2015) explored whether there was convergence among Indian districts, and they found a weak conditional convergence but an absolute divergence. Most recent studies found that the states belong to a different equilibrium state and converge to other steady states owing to variations in beginning circumstances, and the studies suggest that there is a need for absolute convergence (Ghosh, 2008; Bandyopadhyay, 2011; Ghosh et al., 2013; Mishra and Mishra, 2018; Hembram and Haldar, 2019). Another way of putting it is that different equilibria are determined by uneven economic forces, while a different steady-state economy pertains to the income generated by those resources. Similarly, Hembram et al. (2019) investigated the ‘club convergence’ in income across 15 Indian states from 1982 to 2014 using the Markov chain and stochastic kernel approaches. They found that the club convergence in-state distribution and the convergence in GDP per capita re-established the idea of the “low-level equilibrium trap” associated with poor human capital investment. Other empirical research explores how health spending has converged among Indian States. Research suggests that various non-income-related variables, such as technical advancement, socio-economic disparities, demographic variations, and political institutions, are also responsible for the significant disparity (Apergis and Padhi, 2013; Youkta and Paramanik, 2020). Additionally, only a few research has examined the club convergence of per capita income and HDIs for Spanish provinces (Montañés et al., 2018).

Some works of the literature analysed the convergence of HDIs across nations; however, no study focused on Indian states and union territories (UTs). Hence we try to fill this gap by examining the intersection of human development across Indian states and UTs. It is crucial to investigate India’s HDI for several reasons. Firstly, India has one of the largest and fastest-growing economies in the world, with an annual growth rate of 8.9% in its GDP since 1990 (WDI, 2021). India is the world’s second-most populous country, accounting for 17.7% of the world’s population. However, it has shown a disappointing level of human development, with an HDI score of 0.645, placing it at 131 among the 189 nations (UNDP, 2019). Second, India does have a shorter average life expectancy, i.e., 69 years and 4 months (UNPD, 2019). Third, there is a problem with equitable access to education in India. The literacy rate in India was about 77.7% (NSO, 2017–18), and according to UNESCO (2019), 35% of the world’s illiterate population resides in India. Improving health, education, and income would ensure a rise in the HDI. Therefore, testing the convergence of the HDI across Indian states and UTs is informative and has policy implications. If Indian states and UTs experience a similar convergence in the HDI, a standard national development policy will be effective. However, if Indian states and UTs experience a different convergence in the HDI, a more nuanced development policy must account for such differences. In this context, the method allows the possibility of club convergence of the HDI across Indian states and union territories.

To our knowledge, no studies have explored the convergence of HDIs across Indian states and UTs using the Phillips and Sul (2007) technique. The paper assesses the relative performance of Indian states and UTs and the comprehension of inter-regional disparity in the HDI by applying the Gini and Theil indices. We also explore the convergence hypothesis using the techniques proposed by Phillips and Sul and kernel density estimators to assess club convergence since the study covers a substantial period during which the country experienced a change in its administrative boundaries. Additionally, we estimate the convergence rate using the Phillips and Sul methodology, which needs to be documented in the existing literature.

We ought to explore the potential convergence across the Indian states and UTs concerning the HDI. The study’s null hypothesis is that the HDI does not converge across Indian states and UTs. We will test the null hypothesis by analysing literature and research studies. Our study reveals several significant findings which indicate that the Indian states and UTs have different transition paths in the human development index. Phillips and Sul (2007) formed the club using a clustering algorithm for states with similar transition paths. In the first classification, we find three clubs that are significantly convergent. The Phillips and Sul (2007) approach can lead to too many clubs being chosen; in response, they suggest that tests for club merging should be conducted. After evaluating the pattern of the final club, we determined that there are two final clubs. Different clubs converge at different equilibrium positions. This finding has policy implications for the human development index in India. The measures uniform to all states and territories will have a limited impact on the states and territories with different convergence patterns. These clubs must be taken into consideration while formulating Indian human development policies. The studies’ findings may provide policymakers insight into achieving horizontal equity across Indian states.

## Data and sources

We have used data from the Global Data Lab, which is maintained by the Institute for Management Research at Radboud University in the Netherlands. The study used annual data of the HDI for 36 Indian states and UTs from 1990 to 2019. The Global Data Lab has provided information on the HDI at the national and subnational levels since 1990. The database contains data for 186 countries and 1783 subnational regions from 1990 to 2019 (Smits and Permanyer, 2019). The values of the four indicators at the subnational level of India were made available from statistical offices and the Area Database of the Global Data Lab, which contains indicators aggregated from household surveys and census datasets. The value for the missing year is estimated by interpolation and extrapolation from actual data. The four indicators are constructed so that their population-weighted national averages are equal to their national values in the United Nations Development Programme-Human Development Index database. The methodology used for HDI construction is the same as the United Nations’ (2019) methodology for constructing national HDI.

### Procedure for estimating HDI

As follows, HDI is a simple arithmetic mean of all three primary indices (UNDP, 2019):

1 $${\rm {HDI}}_{it} = \sqrt {{\rm {HI}}_{it} \ast {\rm {EI}}_{it} \ast {\rm {II}}_{it}}\, t = 1,\,2,\, \ldots \, \ldots \,..,N$$

where HI, EI, and II mean a health index, an education index, and an income index observed across *i* = 1, 2, …, *N* and *t* = 1, 2, …, *N*, which denote the number of Indian states and union territories, and sample size, respectively. The HI depends on life expectancy at birth (LEB) and its construct is as follows:

2 $${\rm {HI}}_{it} = \frac{{{\rm {LEB}}_{it} - {\rm {LEB}}_{min}}}{{{\rm {LEBMax}} - {\rm {LEBmin}}}}$$

where LEBmin and LEBmax are being 20 and 85 years old, respectively. EI denotes an education index that can be defined as follows:

3 $${\rm {EI}}_{it} = \frac{{{\rm {MYSI}}_{it} + {\rm {EYSI}}_{it}}}{2}$$

where MYSI_*it*_ is the mean years of schooling index and EYSI_*it*_ is the expected year of schooling index. These indexes are obtained as follows:

4 $${\rm {MYSI}}_{it} = \frac{{{\rm {MYS}}_{it}}}{{15}}$$

5 $${\rm {EYSI}}_{it} = \frac{{{\rm {EYS}}_{it}}}{{18}}$$

The maximum for the mean years of schooling (MYS) is 15. The maximum for expected years of schooling (EYS) is 18, which is equivalent to achieving a master’s degree in most countries. Societies can exist without formal education, which justifies a 0-year education requirement (UNDP, 2019). MYS_*it*_ *and* EYS_*it*_ are the number of Indian states and union territories that have achieved and sample size, respectively.

Finally, II is an income index that can be defined as follows:

6 $${\rm {II}}_{it} = \frac{{{{{\mathrm{ln}}}}\left( {{\rm {GNI}}_{it}} \right) - {{{\mathrm{ln}}}}\left( {100} \right)}}{{{{{\mathrm{ln}}}}\left( {75,000_{it}} \right) - {{{\mathrm{ln}}}}\left( {100} \right)}}$$

where GNI_*it*_ is the gross national income per capita of *i*th states at period *t*.

Now classified into four groups after being introduced in 2014 (UNDP, 2019), they are as follows: 0.00 < HDI < 0.550 = Low level; 0.550 < HDI < 0.699 = Medium level; 0.700 < HDI < 0.799 = High level, and 0.800 < HDI < 1.00 = Very high level.

## Background of the convergence

Convergence analysis has been a vital study topic in the economics literature due to the policy importance of determining whether poorer regions can reach the same level of the outcome as wealthier ones (Barro and Sala-I-Martin, 1992, 2003; Bernard and Jones, 1996; Nixon, 1999). The concept of convergence is derived from Solow’s neoclassical growth model (Solow, 1956; Swan, 1956). Considering the Inada (1963) condition, Solow’s essential assumption is that as the marginal product of capital or labour approaches infinity, capital or labour goes to zero, and vice versa. Baumol (1986) was the first to introduce the idea of beta (*β*) convergence, which denotes a negative connection between the growth rate of an interesting variable and its starting level. It was further developed by Barro and Sala-i-Martin (1992). Although many different hypotheses have been proposed in this context, absolute convergence, sigma convergence, conditional convergence, club convergence, and stochastic convergence stand out as various kinds of convergence (Panopoulou and Pantelidis, 2009; Morales-Lage et al., 2019). Absolute convergence, also known as *β* -convergence, is the process through which lag regions grow more rapidly than advanced regions (Barro and Sala-i-Martin, 1992) and eventually catch up to them (Mankiw et al., 1992; Islam, 1995; Sala-i-Martin, 1996). It can be assessed by evaluating the nonlinear regression that shows an inverse correlation between the growth rate and its initial level (Baumol, 1986). De Long (1988) and Quah (1993a) have criticised β-convergence and demonstrated how this technique could lead to spurious levels of convergence. In other words, testing may demonstrate convergence even without it.

On the other hand, Sigma convergence refers to a reduction over time in the cross-section variation of the relevant variables’ natural logarithm. Usually, variance is measured using the sample standard deviation (Barro and Sala-i-Martin, 1990). Beta convergence is a necessary but insufficient condition for sigma convergence (Sala-i-Martin, 1996). This is imperative to test the σ convergence alongside β- convergence (Lichtenberg, 1994; Young et al., 2008). conditional convergence indicates convergence if the countries have specific characteristics (Morales-Lage et al. 2019).

In contrast, club convergence refers to the tendency of a group of economies to converge to the same steady state when their circumstances and structural features (such as technology, desires, and political systems) are similar (Morales-Lage et al., 2019). The last type of convergence is *stochastic convergence*. According to Quah (1993a), looking at how long shocks persist on the variable would be interesting. A time-series idea of stochastic convergence is presented (Carlino and Mills, 1993, 1996). According to stochastic convergence, transitory shocks in the per capita outcome logarithm relative to the sample average may be expected.

### Methodology

In this paper, we investigate the convergence hypothesis for the human development index across 36 Indian states/union territories using the approach suggested by Phillips and Sul (2007, 2009). The “log *T*-test,” often known as the Phillips and Sul (2007) approach, is a process for evaluating potential, convergence, divergence, and club convergence. It includes comparing the alternative hypothesis that there is convergence to the null hypothesis that there is no convergence. To accept or reject the null hypothesis, Phillips and Sul (2007) provide the crucial value of −1.65. Suppose the estimated log (*t*) statistics value for the entire sample is less than the critical value of −1.65. In that case, we reject the null hypothesis and accept the alternative that there is convergence. Next, we can form the club using a clustering algorithm after confirming the presence of convergence in the model. The clustering algorithm, which allows us to classify states into convergence groups, is briefly discussed in the log *t*-test. Furthermore, we estimate kernel density estimates to determine the convergence club.

### The log *t*-test

The methodology is proposed by Phillips and Sul (2007), to test the convergence hypothesis. The PS model defines as a nonlinear time-varying factor model that applies as

7 $${\rm {HDI}}_{it} = {{\,}^{\rm{g}}}_{it} + a_{it}$$

where HDI_*it*_ is the dependent variable for the human development index observed across *i* = 1, 2, …, *N* and *t* = 1, 2, .., *N*, which denote the number of Indian states & UTs and sample size, respectively. HDI_*it*_ is frequently decomposed into two components: ^*g*^_*it*_, is the idiosyncratic factor that captures individual and time-specific effects, and *a*_*it*_, is the transitory component. Phillips and Sul (2007) transform (7) in a way that common and idiosyncratic components in the panel are separated.

8 $$HD{I_{it}} = \left( {\frac{{{{{\,}^{\rm{g}}}_{it}} + {\alpha _{it}}}}{{{\mu _{it}}}}} \right){\mu _{it}} = {\delta _{it}}{\mu _t},\,for\,all\,i\,and\,t$$

where *μ*_*it*_ is the common factor across the states and *δ*_*it*_ is a time-varying idiosyncratic component that captures individual economic performance distances between the common trend components and HDI_*it*_. The time-varying elements *δ*_*it*_ is modelled in a semiparametric form as

9 $$\delta _{it} = \delta _i + \sigma _{it}\varepsilon _{it},\,\sigma _{it} = \frac{{\sigma _i}}{{{{{\mathrm{log}}}}\left( t \right)t^a}},\sigma _i \,>\, 0$$

where *δ*_*it*_ is fixed, across individuals across *i* = 1, 2, .., *N* and weakly dependent over time *t* a denotes the speed of convergence. Finally, *L*(*t*) is a slowly varying function, for which *L*(*t*) → ∞ as *t* → ∞ for *α* ≥ 0.

Convergence among all states and overall convergence is the hypothesis of relevance form (H_0_:*δ*_*i*_ = *δ* for all *i* with *α* ≥ 0) against the alternative hypothesis of no convergence for a particular state or states (*H*_*a*_:*δ*_*i*_ = *δ* for all *i* with *α* < 0). Then there’s the possibility of general divergence, and sub-panels of states moving to various steady states or club convergence, with divergent states (*H*_*a*_:*δ*_*i*_ ≠ *δ* for some *i* with *α* ≥ 0 or *α* ≥ 0 or *α* *<* 0).

As *μ*_*it*_ is a common element in Eq. (8), it can be scaled out to get the relative transition coefficient, which can assess convergence and long-run equilibrium. *h*_*it*_, for calculating the loading coefficient. *δ*_*it*_ it with respect to the panel average at time *t*. The parameter can be estimated as follows:

10 $$h_{it} = \frac{{{\rm {logHDI}}_{it}}}{{N^{ - 1}\mathop {\sum}\nolimits_{i = 1}^N {{\rm {logHDI}}_{it}} }} = \frac{{\delta _{it}}}{{\frac{1}{N}\mathop {\sum}\nolimits_{i = 1}^N {\delta _{it}} }}$$

if *h*_*it*_→1, *δ*_it_ → *δ*_*i*_, Therefore the variance of *h*_*it*_ should convergence towards unity, the cross-sectional variation should converge to zero and when *T* go toward infinite. Then we have

11 $$H_t = \frac{1}{N}\mathop {\sum}\limits_{i = 1}^N {\left( {h_{it} - 1} \right)^2}$$

The coefficient of assessment and capture of divergent individual behaviour illustrates the relative transition route from common stochastic trends when testing the null of convergence and grouping individuals into convergence clubs in the preceding equation. There are two components to the process. We start by determining whether or not convergence exists. The potential of club convergence is then investigated. The null, according to PS, is convergence, which we evaluate using the following regression model:

12 $$log\left( {\frac{{H_1}}{{H_t}}} \right) - 2logL\left( t \right) = \alpha + \beta logt + \mu _t$$

where for *t* = [*rT*], [*rT*]+1,… .,*T*. with an *r* > 0, starting with *t* = [*rT*], being the integer components *rT* for some fraction *r* > 0, Phillips and Sul (2007) recommend that the *r* value be set at 0.3. Since *β* = 2*α*, *β* coefficient gives a scaled estimation of the speed of convergence parameter an under the null convergence parameter *α*. A one-sided *t*-test of *α* ≥ 0, which is rejected at the 5% significant level if *t*_*b*_ < −1.65, can thus be used to test convergence. Furthermore, *β* assesses the speed of convergence of the relative transition parameter *δ*_*it*_ not only in the sign of the coefficient *β* = 2*α* but also in its magnitude. Hence, the estimate *β* ≥ 2 (*α* ≥ 1) denotes absolute convergence, i.e., convergence to a specific club indicates a level of convergence. This rate of convergence corresponds to conditional convergence, whereas 2 ≥ *β* ≥ 0. Phillips and Sul (2007) recommend employing a four-step club convergence procedure in their empirical use of the log *t*-test to test for convergence.

(i) Order the States and UTs in the sample accordingly to the HDI in the last period.
(ii) Form the core group of k* states by selecting the *k* states with higher HDI to form a sub-group Gk and run a convergence test. Run the log *T* regression for the first *k* units, then select the core group by maximising the value of convergence *t*-statistics subject to the restriction that it is more significant than −1.65.
(iii) Add one state to the core group at a time and run the convergence of log *t*-test. The state is included; if log *tk* is greater than the critical value −1.65, the initial club convergence is obtained.
(iv) For the remaining state, repeat steps (ii) and (iii) in the same group to determine where there is another subgroup that constitutes the convergence club. If no *k* in step 2 satisfies the condition *tk* > the critical value −1.65, then the remaining state does not form any sub-convergence group or unit diverge.

### Kernel density estimator

Kernel density estimates are widely utilised in non-parametric convergence studies. It is useful to describe it as; Let *f* = *f*(*x*) represent the continuous density function of a random variable *X* at a given point *x*, and *x*_1_, …, *x*_*n*_ represent the observations from *f*.

The *k* kernel function is as (Pagan and Ullah, 1999; Rosenblatt, 1956) follows:

13 $${\int}_{ - \infty }^\infty {k\left( y \right){\rm {d}}y = 1\,{\rm {where}}\,k\left( y \right) \ge 0}$$

The general kernel estimator *f*^(*x*) is defined by

14 $$\widehat {f\left( x \right)} = \frac{1}{{hn}}\mathop {\sum}\limits_{i = 1}^n {k\left( {\frac{{X_i - x}}{h}} \right)} = \frac{1}{{nh}}\mathop {\sum}\limits_{i = 1}^n {k\left( {y_i} \right)}$$

Where *y*_*i*_ = *h*^−1^ (*x*_i_−*x*), *n* defines the number of observations in the sample, and *h* is the window width(bandwidth) which is a function of the sample size and goes to zero as *n* → *∞* (Quah, 1993b).

## Results

### Performance of the human development index across the Indian states and union territories

First, we evaluated the relative performance of HDI and its growth rates across all 36 Indian states/union territories during 1990–2019. We also examined how each state’s rankings changed over time on the HDI at various points.

**Fig. 1:**
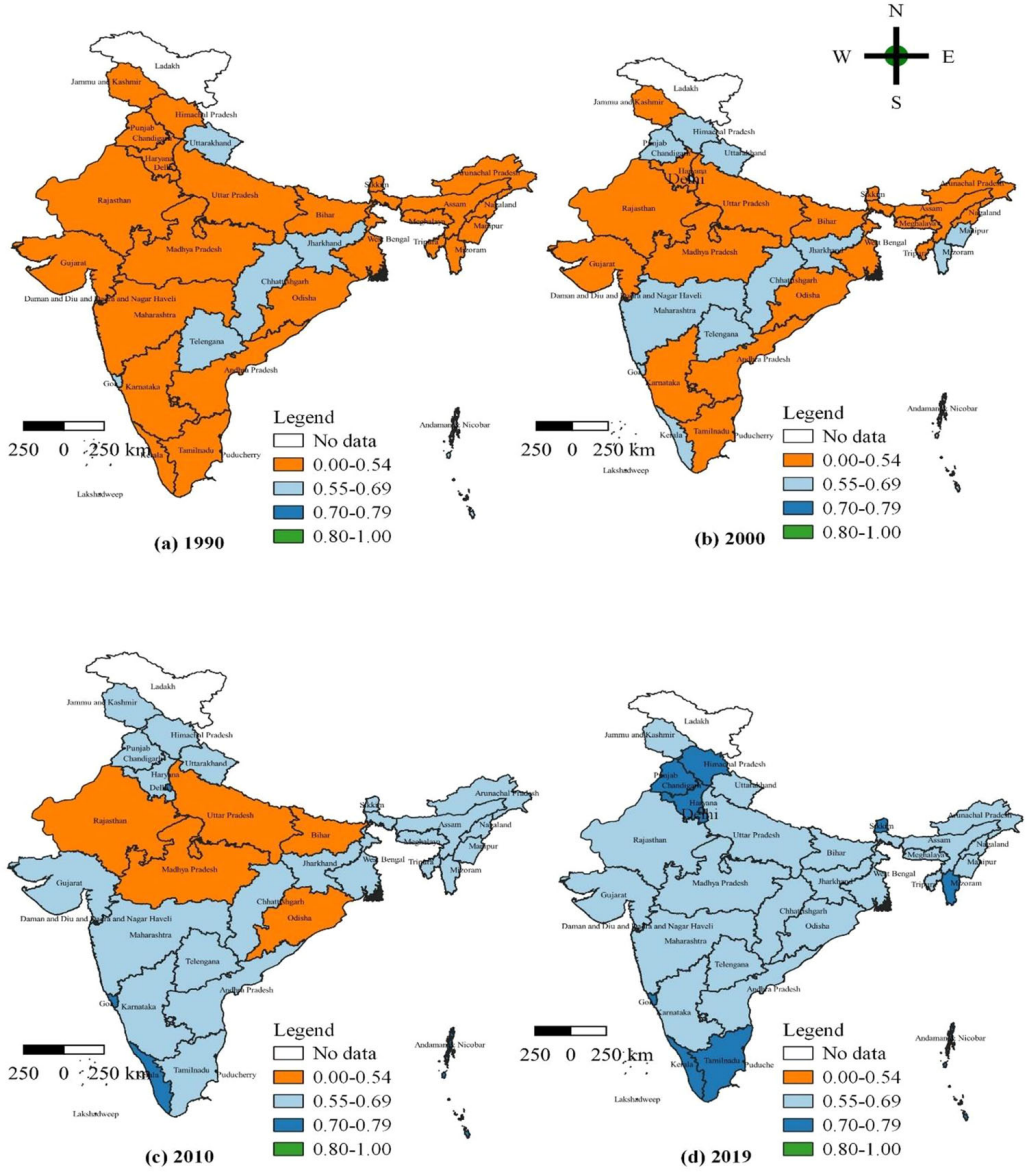
The temporal and spatial evaluation of IHDI in 36 Indian states and union territories: 1990–2019. Source: Authors’ compilation based on data from the Global Data Lab(GDL). The map was developed by the authors using QGIS Version 3.24.0, and the map was cross verified with the India map and its States and Union Territories’ boundaries as shown on the official website of the Survey of India: https://indiamaps.gov.in/soiapp/.

Table 1 summarises the states’ achievements in human development and their growth rates from 1990 to 2019. India’s HDI is 0.429, 0.494, 0.579, and 0.646 for the four representative years 1990, 2000, 2010, and 2019, respectively, with an average annual growth rate of 1.42%, 1.60%, 1.22%, and 1.42%, respectively. From 1990 to 2000, the average yearly growth rate was 1.42%, and from 2000 to 2010, it was 1.60%, which is more substantial growth than the decrease from 2010 to 2019, with an average annual growth rate of 1.22%. From 1990 to 2019, the average annual growth rate was 1.42%. A liner growing degree of human development may be noticed during the research period at the national level. However, the rate of acceleration has slowed in recent years. According to the United Nations Development Programmer’s classification of human development levels, India’s HDI level has risen from a low point in 1990 to a medium point in 2019. We utilised Arch GIS 3.16 software to visualise the 36 states and union territories in 1990, 2000, 2010, and 2019 to examine the spatiotemporal evolution of HDI in India, as shown in Fig. 1.

**Table 1:** Performance of the human development index across the Indian states and union territories.

State/union territories	1990	R1	2000	R2	2010	R3	2019	R4	R1−R4	1990–2000^a^	2000–2010^a^	2010–2019^a^	1990–2019^a^
Andaman and Nicobar	0.683	3	0.694	3	0.707	6	0.741	6	3	0.16	0.19	0.52	0.28
Andhra Pradesh	0.424	30	0.478	30	0.58	27	0.649	27	−3	1.21	1.95	1.26	1.48
Arunachal Pradesh	0.437	29	0.502	28	0.641	17	0.661	24	−5	1.40	2.47	0.34	1.44
Assam	0.411	31	0.488	29	0.567	31	0.613	30	−1	1.73	1.51	0.87	1.39
Bihar	0.378	36	0.436	36	0.514	36	0.574	36	0	1.44	1.66	1.23	1.45
Chandigarh	0.633	6	0.638	7	0.648	14	0.776	2	−4	0.08	0.16	2.02	0.70
Chhattisgarh	0.562	10	0.564	15	0.574	28	0.611	31	21	0.04	0.18	0.70	0.29
Dadra and Nagar Haveli	0.672	4	0.684	4	0.696	7	0.663	23	19	0.18	0.17	−0.54	−0.05
Daman and Diu	0.651	5	0.664	5	0.677	10	0.708	12	7	0.20	0.19	0.50	0.29
Goa	0.552	12	0.614	10	0.737	2	0.763	3	−9	1.07	1.84	0.39	1.12
Gujarat	0.47	23	0.527	24	0.606	25	0.672	21	−2	1.15	1.41	1.16	1.24
Haryana	0.467	24	0.549	19	0.634	21	0.708	12	−12	1.63	1.45	1.23	1.45
Himachal Pradesh	0.479	21	0.589	12	0.667	11	0.725	8	−13	2.09	1.25	0.93	1.44
Jammu and Kashmir	0.493	19	0.528	23	0.64	19	0.688	17	−2	0.69	1.94	0.81	1.16
Jharkhand	0.562	10	0.564	15	0.574	28	0.598	34	24	0.04	0.18	0.46	0.21
Karnataka	0.444	27	0.518	26	0.605	26	0.683	18	−9	1.55	1.56	1.36	1.50
Kerala	0.544	13	0.598	11	0.714	4	0.782	1	−12	0.95	1.79	1.02	1.26
Lakshadweep	0.693	2	0.705	2	0.717	3	0.751	4	2	0.17	0.17	0.52	0.28
Madhya Pradesh	0.406	32	0.46	34	0.538	33	0.603	33	1	1.26	1.58	1.28	1.37
Maharashtra	0.493	19	0.558	18	0.644	16	0.697	15	−4	1.25	1.44	0.88	1.20
Manipur	0.495	18	0.559	17	0.681	9	0.697	15	−3	1.22	1.99	0.26	1.19
Meghalaya	0.456	25	0.477	31	0.62	23	0.656	26	1	0.45	2.66	0.63	1.26
Mizoram	0.525	16	0.569	14	0.686	8	0.704	14	−2	0.81	1.89	0.29	1.02
Nagaland	0.531	15	0.522	25	0.661	12	0.679	20	5	−0.17	2.39	0.30	0.85
Delhi	0.577	9	0.664	5	0.709	5	0.746	5	−4	1.41	0.66	0.57	0.89
Odisha	0.4	34	0.458	35	0.535	34	0.605	32	−2	1.36	1.57	1.38	1.44
Puducherry	0.717	1	0.73	1	0.743	1	0.74	7	6	0.18	0.18	−0.04	0.11
Punjab	0.496	17	0.578	13	0.657	13	0.724	9	−8	1.54	1.29	1.08	1.31
Rajasthan	0.403	33	0.469	32	0.548	32	0.628	29	−4	1.53	1.57	1.53	1.54
Sikkim	0.541	14	0.548	20	0.633	22	0.717	10	−4	0.13	1.45	1.39	0.98
Tamil Nadu	0.471	22	0.542	21	0.646	15	0.709	11	−11	1.41	1.77	1.04	1.42
Telangana	0.622	8	0.627	9	0.638	20	0.669	22	14	0.08	0.17	0.53	0.25
Tripura	0.447	26	0.531	22	0.608	24	0.658	25	−1	1.74	1.36	0.88	1.34
Uttar Pradesh	0.397	35	0.463	33	0.535	34	0.594	35	0	1.55	1.46	1.17	1.40
Uttarakhand	0.629	7	0.63	8	0.641	17	0.683	18	11	0.02	0.17	0.71	0.28
West Bengal	0.44	28	0.505	27	0.572	30	0.641	28	0	1.39	1.25	1.27	1.31
India	0.429	–	0.494	–	0.579	–	0.646	131^b^	–	1.42	1.60	1.22	1.42

Sources: UNDP, Global Data Lab; Authors' own calculation.

*R* Rank.

^a^Growth rate of HDI in percentage.

^b^Rank of UNDP.

As shown in Fig. 1, India’s HDI has developed in the following manner. In 1990, all states and union territories had low levels of development, which were denoted by the mustard-yellow colour, except for 12 states and union territories with a medium level of development, denoted by the dashed blue. These 12 states are Andaman and Nicobar, Chandigarh, Chhattisgarh, Dadra and Nagar Haveli, Daman and Diu, Goa, Jharkhand, Lakshadweep, Delhi, Puducherry, Telangana, and Uttarakhand. In 2000, Himachal Pradesh, Kerala, Maharashtra, Manipur, Mizoram, and Punjab were classified as medium-development states denoted by a dashed blue. Lakshadweep and Puducherry were classified as higher-development states (which denoted blue colours), while the remaining states had low levels of development, which were indicated by a mustard-yellow colour. In 2010, the states of Bihar, Madhya Pradesh, Odisha, Rajasthan, and Uttar Pradesh were still developing slowly, as indicated by the mustard yellow. Andaman and Nicobar, Goa, Puducherry, Kerala, Lakshadweep, and Delhi have recorded a higher degree of development, shown by the colour blue. In contrast, the other states were categorised as medium-development states, which were indicated by a dash of blue.

From 2010 to 2019, the human development levels of most states and union territories improved considerably, moving from the medium development level represented by dashed blue in most states to the high development level represented by blue in most states. The following states had high levels of human development in 2019: Andaman and Nicobar, Chandigarh, Daman and Diu, Goa, Lakshadweep, Delhi, Puducherry, Himachal Pradesh, Haryana, Kerala, Mizoram, Punjab, Sikkim, and Tamil Nadu. The remaining states are at a medium level, as indicated by dashed blue. The disparity in development across areas is shrinking across the country. However, there are no such Indian states or union territories that have attained such a very high degree of human development.

Based on the performance of HDI, we tried to determine the level of HDI in terms of state-level rank change from R1 in 1990 to R4 in 2019 (Table 1). A negative deviation indicates that states have improved their ranking due to higher performance of HDI (R4−R1, negative sign). Andhra Pradesh, Arunachal Pradesh, Chandigarh, Assam, Goa, Gujarat, Haryana, Himachal Pradesh, Jammu and Kashmir, Karnataka, Kerala, Odisha, Maharashtra, Manipur, Meghalaya, Mizoram, Delhi, Punjab, Rajasthan, Sikkim, Tamil Nadu, and Tripura these states are improved their ranking due to higher performance of HDI. In contrast, Andaman and Nicobar, Chhattisgarh, Dadra, and Nagar Haveli, Daman and Diu, Jharkhand, Lakshadweep, Madhya Pradesh, Nagaland, Puducherry, Telangana, and Uttarakhand have all fallen their rank from R1 to R4 due to their low performance on the HDI (R4−R1, positive sign). On the other hand, with an unsatisfactory HDI performance, West Bengal, Bihar, and Uttar Pradesh have yet to improve their rankings and have remained at R1 in 1990 to R4 in 2019, respectively (R4−R1, equivalent). States that have shown progress in state-level rank changes and those that have not yet done so must raise their HDI ranks to help the overall Indian HDI. The advancement achieved by the states that have a way to go would boost India’s HDI.

A simple analysis of the annual growth rates of different states was conducted to assess HDI’s growth dimension. Table 1 shows the states’ annual growth rates for the four periods, which we have divided into the sample. According to the findings, most states had a moderate growth rate from 1990 to 2000. Between 2000 and 2010, the rate of growth accelerated significantly. From 2010 to 2019, the growth rate was diminished. The growth rates of Andhra Pradesh, Arunachal Pradesh, Assam, Bihar Goa, Gujarat, Haryana Himachal Pradesh, Jammu and Kashmir, Karnataka, Kerala, Madhya Pradesh, Maharashtra, Manipur, Meghalaya, Mizoram Odisha, Punjab, Rajasthan, Sikkim, Tamil Nadu, Uttar Pradesh Tripura and West Bengal was modest up to 2000. During the period 2000–2010, the growth rate accelerated. After that, the annual growth rate diminishes. However, the growth rates of Chandigarh, Chhattisgarh, Dadra and Nagar Haveli, Daman and Diu, Jharkhand, Lakshadweep, Nagaland, Delhi, Puducherry, Uttarakhand, and Telangana were much more moderate during the sample period although they have placed good position in state HDI ranking.

India’s degree of human development has increased over the 29 years from 1990 to 2019. While much progress has been made in human development over the last three decades, there are still many significant differences between regions of India. There are numerous issues with the development process, including a significant divide between regional development and development that needs to be balanced or sufficient. All indicators indicate that the socio-economic development of India’s various states is merging at a specific rate. However, integration appears to be proceeding at a snail’s pace.

### Inequality trend of the human development index

The HDI achievement of 36 Indian states and union territories is being analysed to ascertain the evolution of inequality from 1990 to 2019. Except for 2015–2019, we examined HDI at 5-year intervals from 1990 to 2015. We used four well-known variables, including standard deviation (SD), coefficient of variation (COV), and two major inequality indices, the Gini index and the Theil index (Theil, 1967), to create a dynamic picture of inequality in the HDI (Pillarisetti, 1997). We measure inequality using all available methods, irrespective of whether unweighted or weighed states based on population size are used to estimate the population share of states.

Based on the data from 36 Indian states during the study period 1990–2019, human development has steadily progressed on average. Table 2 shows that, on average, at the state level, HDI has risen from 1990 to 2019. The states exhibit extraordinary human development. Sigma convergence is widely discussed in economics literature regarding standard deviation and coefficient variation (CV, the ratio of standard deviation to mean distribution), with the implicit assumption that examined variable’s steady-state level and time trend for all states. Therefore, sigma convergence only depicts the diminishing cross-sectional dispersion. When the sigma of convergence declines, human development exhibits a trend toward convergence. As a result, we employ the sigma convergence of the HDI regional distribution from 1990 to 2019. In Table 2, the standard deviation decreases over time, indicating that the level of human development is convergent. All indicators suggest that the socio-economic development of India’s different states is converging at a certain speed. However, convergence appears to be progressing at a slower rate.

**Table 2:** Inequality trends of the human development index across Indian states and union territories (1990–2019), *N* = 36.

HDI (Population-Unweight)						HDI (Population-Weight)				
Year	Mean	SD	Cov	Gini	Theil	Mean	SD	Cov	Gini	Theil
1990	0.514	0.094	0.183	0.102	0.0158	0.452	0.093	0.206	0.115	0.0039
1995	0.538	0.086	0.161	0.090	0.0123	0.480	0.086	0.179	0.101	0.0034
2000	0.559	0.077	0.138	0.077	0.0092	0.507	0.077	0.151	0.086	0.0029
2005	0.599	0.074	0.124	0.070	0.0074	0.547	0.078	0.142	0.077	0.0027
2010	0.632	0.062	0.098	0.055	0.0048	0.584	0.072	0.124	0.057	0.0023
2015	0.662	0.055	0.082	0.046	0.0034	0.625	0.065	0.104	0.046	0.0017
2019	0.681	0.055	0.081	0.046	0.0032	0.646	0.063	0.098	0.047	0.0015

Author’s own estimation from Global Data Lab.

The Gini and Theil inequality indices for the HDI across all states and UTs (Both population-unweighted and population-weighted) are seen in Table 2. From 1990 to 2019, both inequality indices showed positive growth. The distribution of human development has become more equitable between states. Inequality has decreased significantly during the last 29 years. However, from 1990 to 2019, the inequality trend in the HDI of India’s states and UTs indicates a slight fall and weak convergence tendency. The Gini index (population-weighted) does not fluctuate significantly. It has increased little recently in 2019 and declined when the oval trend coincides with the weak convergence.

Several conclusions can be drawn from the descriptive analysis. All the states do not appear to have a distinct convergence pattern due to the large gap between them at the beginning of the sample. This outcome indicates the presence of diverse behaviour clubs. More appropriate methodologies should be employed to determine whether all states and UTs have a convergence process. In the following section, a question will be addressed.

### Findings of Log *T*-test

The Phillips and Sul (2007) methodology, commonly known as the “log *T*-test,” was used to examine potential convergence, club convergence, and divergence in the HDI’s observation distribution variation across a sample of Indian states and UTs. In the full sample, the log (*t*) statistics value for the entire sample is −15.2339, less than the critical value of −1.65, according to Table 3. As a result, here we rejected the null hypothesis of HDI convergence at the 5% significance level, which indicates that the Indian states and UTs have different transition paths in the HDI. The finding shows that the HDI of Indian states and UTs do not follow a single development path. As a result, it is conceivable to have a heterogeneous equilibrium with distinct outcomes. Phillips and Sul (2007) formed the club using a clustering algorithm for states and UTs with similar transition paths. In the first classification, the findings indicated the existence of three clubs: club 1 contains 5 states, club 2 has 24 states, and club 3 contains 7 states. The log (*t*) values for these clubs are 3.860, 5.816, and 9.727, respectively. Each value is more than the critical value (−1.65). We are unable to reject the null hypothesis.

**Table 3:** Human development index (HDI) convergence.

Initial classification	States and Union Territories	*β* coeff	*T*-stat
Full sample	All states and Union Territories	−0.3781	−15.2339^a^
Club 1[5]	Andaman and Nicobar, Goa, Kerala, Lakshadweep, Puducherry	1.559	3.860
Club 2[24]	Andhra Pradesh, Arunachal Pradesh, Chandigarh, Dadra and Nagar Haveli, Daman and Diu, Gujarat, Haryana, Himachal Pradesh, Jammu and Kashmir, Karnataka, Maharashtra, Manipur, Meghalaya, Mizoram, Nagaland Delhi, Punjab, Rajasthan, Sikkim, Tamil Nadu, Telangana, Tripura, Uttarakhand, West Bengal	0.261	5.816
Club 3[7]	Assam, Bihar, Chhattisgarh, Jharkhand, Madhya Pradesh, Odisha, Uttar Pradesh	2.270	9.727
*Merge of clubs*			
Club 1 + 2 [29]		0.2227	4.9388
Club 2 + 3[7]		−0.4874	−10.4350^a^
Final clubs’ classifications after merge			
Club 1[29]	Andaman and Nicobar, Andhra Pradesh, Arunachal Pradesh, Chandigarh, Dadra and Nagar Haveli, Daman and Diu, Goa, Gujarat, Haryana, Himachal Pradesh, Lakshadweep, Jammu and Kashmir, Karnataka, Kerala, Maharashtra, Manipur, Meghalaya, Mizoram, Nagaland, Delhi, Puducherry, Punjab Rajasthan, Sikkim, Tamil Nadu, Telangana, Tripura, Uttarakhand, West Bengal	0.223	4.939
Club 2[7]	Assam, Bihar, Chhattisgarh, Jharkhand, Madhya Pradesh, Odisha, Uttar Pradesh	2.270	9.727

Sources: Global Data Lab.

^a^The value of *t*-statistics is less than the critical value is 1.65 at a 5% level of significance and hence, we reject the null hypothesis of HDI convergence.

**Fig. 2:**
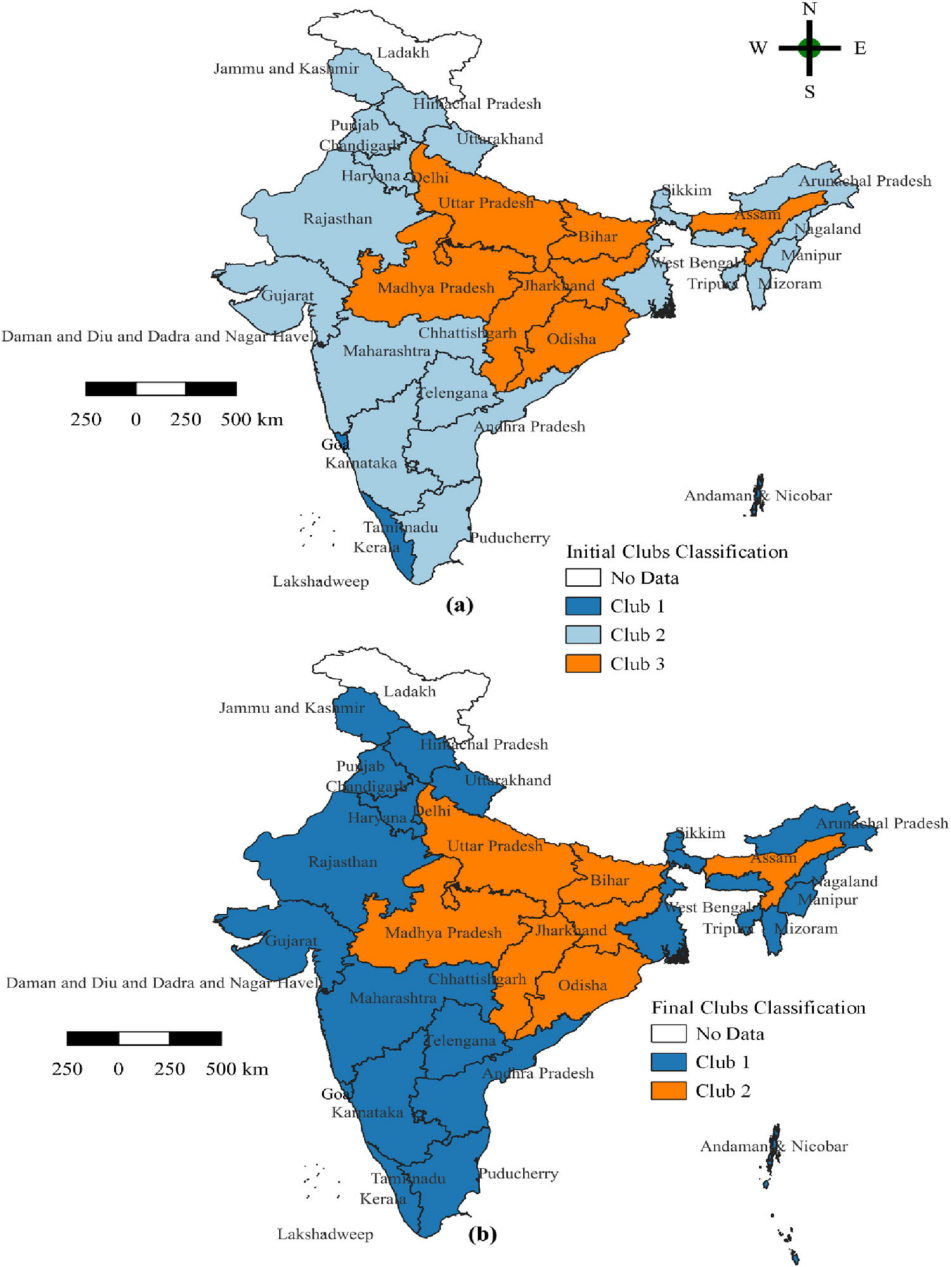
A geographical representation of estimated clubs for the human development index across the Indian states and union territories. **a** Initial clubs’ classification. **b** Final clubs’ classification. Source: Authors’ compilation based on data from the Global Data Lab(GDL). The map was developed by the authors using QGIS Version 3.24.0, and the map was cross verified with the India map and its States and Union Territories’ boundaries as shown on the official website of the Survey of India: https://indiamaps.gov.in/soiapp/.

The first convergence club (club 1) of HDI consists of those with a higher level of development, namely the Andaman and Nicobar Islands, Goa, Kerala, Lakshadweep, and Puducherry. The second convergence club (club 2) shows a medium rate of HDI and more homogeneous behaviour. It includes states like Andhra Pradesh, Arunachal Pradesh, Chandigarh, Dadra and Nagar Haveli, Daman and Diu, Gujarat, Haryana, Himachal Pradesh, Jammu and Kashmir, Karnataka, Maharashtra, Manipur, Meghalaya, Mizoram, Nagaland, Delhi, Punjab, Rajasthan, Sikkim, Tamil Nadu, Telangana, Tripura, Uttarakhand, and West Bengal. Finally, the third convergence club (club 3) comprises Assam, Bihar, Chhattisgarh, Jharkhand, Madhya Pradesh, Odisha, and Uttar Pradesh, which have a lower HDI and are homogeneous groups. Figure 2 shows how the convergence club for the HDI is spread across Indian states and UTs. Figure 2a indicates a clear geographical division between the states and UTs included in these clubs. The region is divided into three clubs. Club 1 represents blue, club 2 represents dashed blue, and club 3 represents mustard yellow (see Fig. 2a).

Furthermore, Phillips and Sul (2009) recommend exaggerating rather than underestimates club convergence rather than the actual number. The clustering method is used between the clubs to see if there is any evidence in favour of clubs merging into larger clubs or between clubs. The result indicates that the merging of the initial clubs 1 + 2 has a log(t) value of 4.9388, which is greater than the critical value of −1.65 but statistically insignificant, indicating that the merged clubs have not converged. On the other hand, clubs 2 + 3, with a log (*t*) value of −10.4350, less than the critical value of −1.65, show that they are statistically significant, indicating convergence among the individuals in each merged club. Finally, we determine the final club classification by performing the club merging test. After evaluating the pattern of the final club, we decided that there is a distinct pattern of clubbing among the states. Club 1 is the largest, with 29, whereas Club 2 has only seven (Table 3). In terms of HDI, the high and moderate-performance states are merged. The result of the merger reveals the two final convergence clubs.

Club 1 is the largest, containing 29 states with log (*t*) statistics of 4.939, which is greater than the critical value (−1.65). Club 2 consists of only seven states with a log statistics log (*t*) of 9.727, greater than the critical value (−1.65). In the final, the first HDI convergence club (club 1) comprises twenty-nine states with a high degree of development. In contrast, club 2 comprises seven states with a low level of development. Assam, Bihar, Chhattisgarh, Jharkhand, Madhya Pradesh, Odisha, and Uttar Pradesh are the same as estimated in the initial club 3 and final club 2. Moreover, it is represented in Fig. 2b as the outcome of the club convergence of the HDI. This analysis allows us to investigate whether regions with higher levels of HDI display a different pattern from those with lower levels of development. Once again, the region is divided into two clubs. In the final clubs, club 1 is denoted by the blue colour, and club 2 is indicated by the mustard-yellow colour (see Fig. 2b).

Nevertheless, our findings indicated the presence of two clubs. Again, as shown in Table 4, the convergence rate varies between the two HDI clubs. Convergence occurs at a rate of 0.112% for club 1 and 1.135% for club 2. Club 2 is increasing faster than Club 1, indicating that states with a lower HDI are growing faster than states with a higher HDI. The occurrence of different convergence routes among the states demands special consideration in any regional agreement on the HDI.

**Table 4:** Speed of convergence.

Final clubs	Human development index (HDI)
Club 1	0.112
Club 2	1.135
Average value of club 1 and club 2	0.624

The speed of convergence is estimated through, specially *β* = 2*α.*

### Distribution dynamics; Kernel density estimator

**Fig. 3:**
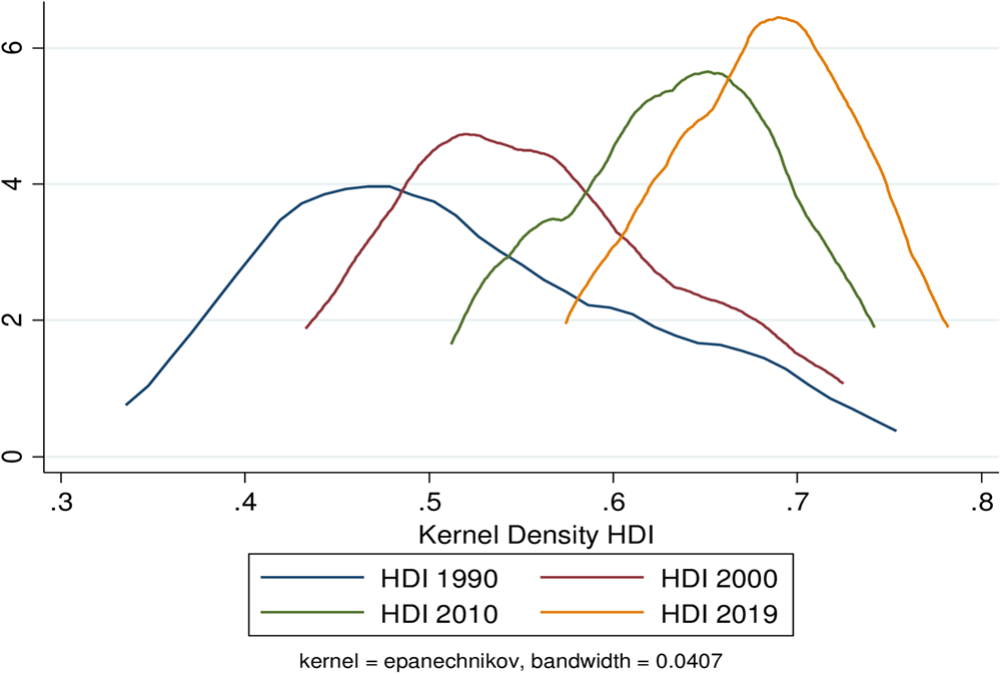
The kernel density distribution for the human development index across Indian states and union territories, 1990–2019.

Density estimates have been obtained using kernel density estimators and displays of normalised data for 1990, 2000, 2010, and 2019 (Fig. 3). Condition patterns encapsulate these estimates, revealing the underlying structure of distribution dynamics and convergence consistency across states. The non-parametric analysis demonstrates that there has been evidence of clubbing, with some states clustered at higher levels of human development and others clustered at lower levels. However, compared to recent years in 2019, the distribution in 1990 was extensively dispersed and scattered. We also see several peaks across multiple endogenously classified states. In 1990, the kernel plot may have revealed a stratification distribution, suggesting that different groups exist. However, in 2000 and 2010, it became polarised and showed a twin-peaked distribution, which indicates the possibility of convergence to a particular steady state. Nevertheless, the highest peak in 2019 occurred immediately after the mean distribution, showing a trend toward a unimodal assemblage around the mean distribution.

## Discussion and conclusion

The paper evaluates the relative performance of Indian states/union territories and understanding inter-regional inequality in the HDI. We also examined the convergence hypothesis by employing the methodology proposed by Phillips and Sul and kernel density estimators to identify club convergence. The analysis is very relevant for India, a country that has made enormous economic and social growth since 1990, and there has been a substantial increase in the HDI. Some states have a low to medium level of development, while others have a medium to a high level of development. Although there has been a significant improvement in human development over the last three decades, we still face enormous inequities. There are various issues with the development process, such as a substantial gap between regional growth and development that needs to be balanced and sufficient. Given considerable interstate variation across all states, we have examined the convergence hypothesis of the HDI. The convergence method employed in this paper allows us to identify regional club convergence by accounting for the HDI in a nonlinear time-varying framework. Our result shows that we did not find evidence in the whole sample, which implies that the selected sample of states is not converging to a single steady state but rather finding the existence of club convergence. The result of estimating the convergence club endogenously identified two final clubs for the HDI.

In particular, we also test the club convergence hypothesis through a non-parametric test. The kernel distribution estimation provides a clear picture of states’ stratification, polarisation, and unimodal distribution regarding HDI overtimes. The kernel plot shows that the distribution is a full mean value of HDI, indicating that states are converging to a common steady state or approaching a unimodal distribution. We find evidence of club convergence on the convergence hypothesis through club specification. The finding of the two convergence clubs for HDI suggests different forces of development and inequality. This finding has policy implications for the HDI in India. The measures uniform to all states and territories will have a limited impact on the states and territories with different convergence patterns. These clubs must be taken into consideration while formulating Indian human development policies. The studies’ findings may provide policymakers insight into achieving horizontal equity across Indian states. The study also suggests that regional development and inequality reduction should be prioritised in light of each clustered state’s unique convergence path for the HDI from the policy perspective.

The Indian government should help accelerate coordinated development across states and create a new regional coordinated development strategy that lays out new standards for bridging India’s regional development gap. India’s regional coordinated development strategy should be refocused on coordinating human development across borders, prioritising minimising regional inequalities in human development and well-being. This approach, including appropriate HDI policies, could help governments improve their overall economic and social development and achieve convergence to a common steady-state level of HDI. Although the HDI has improved and enhanced, the study found that specific issues still need to be addressed. For instance, future research could focus on the spatial clustering of a particular area and its reasons.

## Data Availability

The study analysed the current datasets that are publicly available from the Global Data Lab (https://globaldatalab.org/), which is maintained by the Institute for Management Research at Radboud University, the Netherlands. Data will be made available on request.
